# Genomic insights into *bla*_AFM_-positive carbapenem-resistant *Pseudomonas aeruginosa* in China

**DOI:** 10.3389/fmicb.2025.1546662

**Published:** 2025-05-09

**Authors:** Huimin Lu, Chuanjun Zhang, Buhui Zhao, Yan Li, Shangshang Qin

**Affiliations:** ^1^Department of Pharmacy, Henan Provincial People's Hospital, Zhengzhou, China; ^2^XNA Platform, School of Pharmaceutical Sciences, Zhengzhou University, Zhengzhou, China; ^3^Key Laboratory of Advanced Drug Preparation Technologies, Ministry of Education, Zhengzhou University, Zhengzhou, China

**Keywords:** patients, carbapenem resistance, *Pseudomonas aeruginosa*, *bla*_AFM_, genomics

## Abstract

Carbapenem-resistant *Pseudomonas aeruginosa* (CRPA) poses a global threat; however, the epidemiological characteristics and clinical significance of *bla*_AFM_-positive CRPA strains in China remain unclear. In this study, continuous surveillance was conducted from 2018 to 2022 in a hospital in Henan Province, China, and the genomic characteristics of *bla*_AFM_-positive CRPA were elucidated. We characterised the genetic features of *bla*_AFM_-positive CRPA isolates by antimicrobial susceptibility testing, conjugation assays, whole-genome sequencing, large-scale comparative genomics, and bioinformatic analyses. Among 628 CRPA isolates, one *bla*_AFM_-positive multidrug-resistant (MDR) strain, PA19-3158 (ST1123), was identified, with the *bla*_AFM-1_ gene located on a novel 518,222 bp megaplasmid. Additionally, big data analysis revealed the genomic characteristics of *bla*_AFM_-positive CRPA across China. A total of three different *bla*_AFM_ gene variants were identified among these isolates, namely *bla*_AFM-1_ (44.12%), *bla*_AFM-2_ (52.94%), and *bla*_AFM-4_ (2.94%). Our findings identified ST463 as the dominant clone among *bla*_AFM_-positive CRPA in different regions of China, with some *bla*_AFM_-positive CRPA isolates from these regions exhibiting high genetic similarity. Notably, all *bla*_AFM_-positive CRPA isolates carried multiple antibiotic resistance genes (ARGs), with approximately 38% co-harboring the carbapenem-resistant gene *bla*_KPC-2_ and approximately 47% co-harboring the tigecycline-resistant gene *tmexCD-toprJ*. Correlation analysis underscored the significant role of mobile genetic elements in facilitating *bla*_AFM_ gene transfer. These results highlight the critical need for continuous surveillance of *bla*_AFM_-positive CRPA in clinical settings to mitigate potential risks.

## Introduction

Carbapenem-resistant *Pseudomonas aeruginosa* (CRPA) exhibits extensive drug resistance, poses significant challenges in clinical treatment, and is associated with high mortality rates in infected patients (Zhu et al., 2023; Saravanan et al., 2023). As such, CRPA has been classified as a “critical priority” pathogen for antibiotic development by the World Health Organization (WHO). The resistance of *P. aeruginosa* to carbapenem antibiotics is primarily mediated by several mechanisms: overexpression of the MexAB-OprM efflux pump, hyperproduction of AmpC β-lactamase, inactivation of OprD porins, and production of carbapenemases (Gray et al., 2023; Livermore, 2002). Although carbapenemase-producing *P. aeruginosa* constitutes a smaller proportion of CRPA isolates, this proportion has been steadily increasing in recent years (Reyes et al., 2023). Among these carbapenemases, metallo-β-lactamases (MBLs) are the most prevalent in CRPA (Yoon and Jeong, 2021). The treatment of infections caused by MBL-producing CRPA is particularly challenging, as MBLs confer resistance to most β-lactam antibiotics (Jean et al., 2019).

Historically, NDM was considered the sole member of the subclass B1b MBL. However, in 2018, a novel subclass B1b MBL, *Alcaligenes faecalis* metallo-β-lactamase-1 (AFM-1), was first identified in *Acinetobacter faecalis* isolates in China (Li et al., 2022). Since then, AFM-1 has been detected in various bacterial species, including *P. aeruginosa*, *P. putida*, and *Bordetella trematum* (Li et al., 2022; Zhang et al., 2021). Furthermore, as AFM-1 continues to evolve, multiple variants such as AFM-2, AFM-3, and AFM-4 have been identified in *P. aeruginosa* (Chen et al., 2022). However, despite its emergence, reports on bacteria producing this newly discovered MBL remain scarce, and comprehensive studies on AFM-producing pathogens are still lacking.

To further enhance our understanding of *bla*_AFM_-positive CRPA isolates, this study conducted a five-year surveillance of *bla*_AFM_-positive CRPA isolates from hospitals in Henan Province, China, systematically analyzing their characteristics. Additionally, through large-scale data analysis, the genomic features of *bla*_AFM_-positive CRPA isolates in China were further elucidated.

## Methods

### Characterization of *bla*_AFM_-positive CRPA isolates in clinical settings

To investigate the presence of *bla*_AFM_-positive CRPA among hospitalized patients, continuous surveillance was conducted from 2018 to 2022 in a hospital in Henan Province, China, with over 10,000 beds. The presence of the *bla*_AFM_ gene was confirmed via PCR, and *bla*_AFM_-positive isolates were subjected to further analysis. This study was approved by the Ethics Committee of Zhengzhou University.

### Antimicrobial susceptibility testing of *bla*_AFM_-positive CRPA isolates

Minimal inhibitory concentrations (MICs) were determined using the broth microdilution technique, and *E. coli* ATCC 25922 was used as the quality control. The panel of antibiotics tested included meropenem, gentamicin, ciprofloxacin, amikacin, aztreonam, colistin, ceftazidime, piperacillin/tazobactam, fosfomycin, ceftazidime/avibactam, and aztreonam/avibactam. Resistance breakpoints were interpreted according to EUCAST v.14.0 criteria for tigecycline and CLSI guidelines for the remaining antibiotics (Marchaim et al., 2014; CLSI, 2018).

### Conjugation experiments

To assess the transferability of the *bla*_AFM_-positive plasmid, a conjugation assay was performed using rifampicin-resistant *Escherichia coli* EC600 and hygromycin-resistant *Klebsiella pneumoniae* YZ6 as recipients. Donor and recipient cultures were mixed at a 1:4 ratio and incubated on LB agar plates at 37°C overnight. Transconjugants were selected on LB agar plates supplemented with meropenem (2 mg/L) and rifampin (300 mg/L) or meropenem (2 mg/L) and hygromycin (200 mg/L). PCR was subsequently employed to confirm the presence of *bla*_AFM_ in the transconjugants.

### Whole-genome sequencing and genome assembly

To characterize the genetic features of the identified *bla*_AFM_-positive CRPA isolates, genomic DNA was extracted using the FastPure bacterial DNA isolation mini kit and quantified with a Qubit 4 Fluorometer. Whole-genome sequencing (WGS) was performed on the Illumina HiSeq 2500 platform with 2 × 150 bp paired-end libraries. *De novo* genome assembly was carried out using SPAdes v.3.14.0 (Bankevich et al., 2012). Additionally, *bla*_AFM_-positive plasmids were extracted using the Qiagen Plasmid Midi Kit and subjected to Nanopore sequencing. The plasmid sequences were assembled using Unicycler v.0.4.8 (Wick et al., 2017). Genome annotation was performed with Prokka v.1.11 (Seemann, 2014).

### Screening of *bla*_AFM_-positive CRPA isolates in retrospective data from China

All *P. aeruginosa* genome sequences available in the National Center for Biotechnology Information (NCBI) genome database as of 2 August 2024 were retrieved (Li et al., 2024). Subsequently, *bla*_AFM_-positive *P. aeruginosa* isolates originating from China were selected for further analysis. Basic information regarding the *bla*_AFM_-positive *P. aeruginosa* isolates, including the region, year of isolation, and accession number, was organized into an Excel spreadsheet for downstream analysis.

### Bioinformatics analysis

ARGs and plasmid replicons were identified using online tools with default parameters (threshold value: 80% identity and coverage) (Bortolaia et al., 2020; Carattoli and Hasman, 2020). Virulence genes were annotated using the Virulence Factor Database (VFDB) (Kleinheinz et al., 2014). Multi-locus sequence typing (MLST) of the isolates was determined using the mlst tool. SNPs in the genomes of *bla*_AFM_-positive CRPAs were detected using snp-dists v.0.7.0.^1^ Phylogenetic trees were constructed using Roary and FastTree based on core single-nucleotide polymorphism (SNP) alignments and further refined with iTOL (Price et al., 2009; Letunic and Bork, 2021). Plasmid comparison maps were generated using BRIG v.0.95 and Easyfig v.2.2.3 (Alikhan et al., 2011; Sullivan et al., 2011).

### Statistical analysis

Statistical analyses and visualizations were conducted using R v4.2.2 (R Foundation for Statistical Computing, Vienna, Austria). Spearman correlation analysis was employed to assess relationships among ARGs, insertion sequences, virulence factors, and plasmid replicons. Clinical data were organized using Microsoft Excel 2013.

### Data availability

Genome sequence data reported in this study have been deposited in the National Center for Biotechnology Information under the accession number NZ_CP140112.1 (pAFM-PA19-3158). All study data are included in the article and/or supporting information.

## Results

### Overview of CRPA isolates

In this study, a single *bla*_AFM-1_-positive CRPA strain, designated PA19-3158, was isolated from a hospital in Henan Province, China. This strain was isolated from the blood sample of a 29-year-old male patient in the ICU. MLST analysis revealed that PA19-3158 belongs to ST1123. MIC testing demonstrated resistance to carbapenems as well as aztreonam, ceftazidime, piperacillin/tazobactam, ceftazidime/avibactam, and aztreonam/avibactam, while retaining susceptibility to ciprofloxacin and colistin (Supplementary Table S1). Additionally, conjugation experiments indicated that the *bla*_AFM-1_ gene in PA19-3158 was non-transferable to *E. coli* EC600 and *K. pneumoniae* YZ6.

### Characterization of *bla*_AFM_-positive plasmids

**Figure 1:**
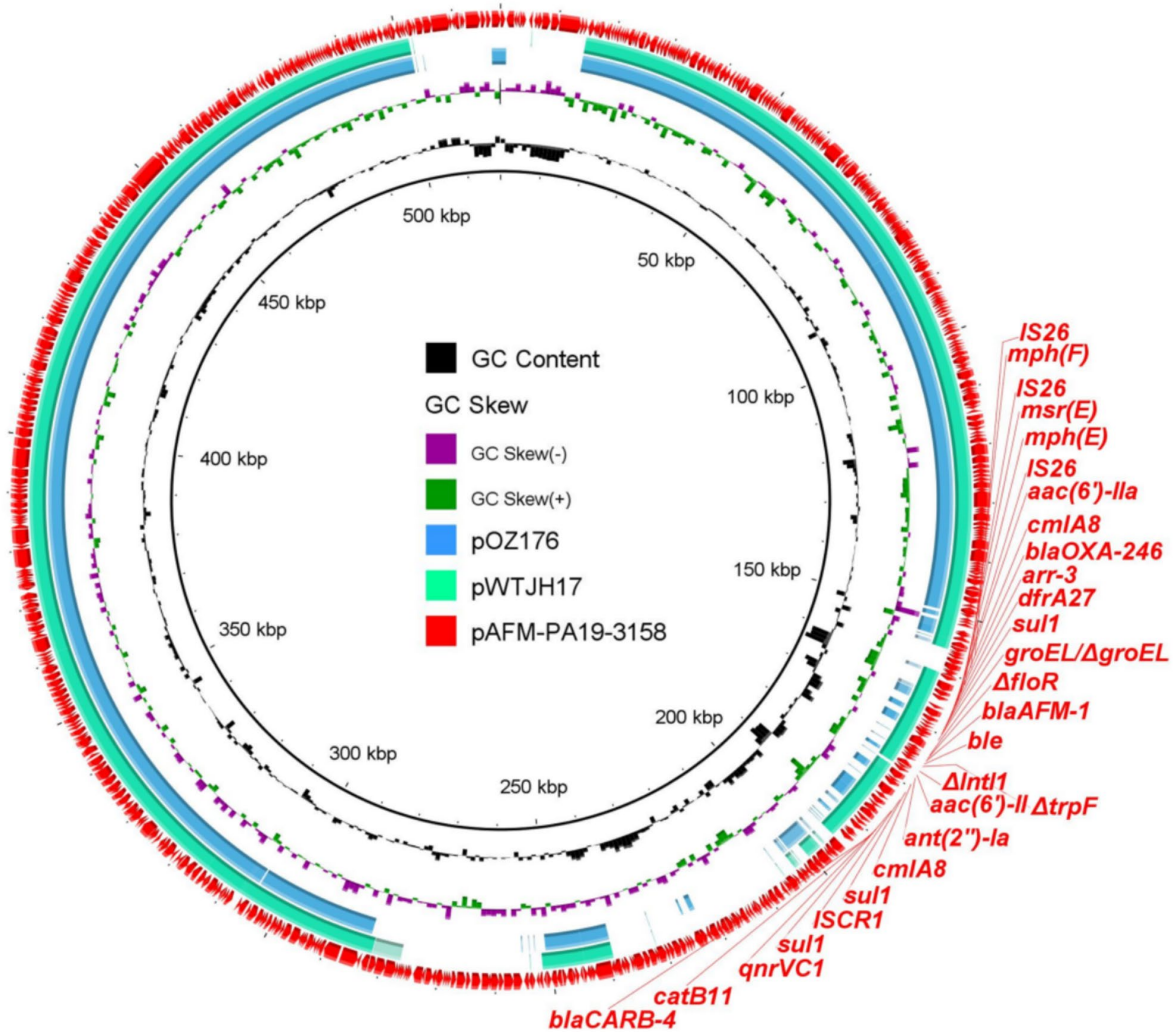
Circular comparison between *bla*_AFM_-bearing plasmids found in this study and the NCBI database. The *bla*_AFM_-bearing plasmid pAFM-PA19-3158 was used as the reference in the outermost ring.

To investigate the characteristics of *bla*_AFM_-positive plasmids, the plasmids carrying *bla*_AFM-1_ from PA19-3158 were extracted and subjected to long-read sequencing using the Nanopore platform. Through the combined analysis of short-read and long-read sequencing data, we identified that the *bla*_AFM-1_ gene is located on a 518,222 bp megaplasmid, with an average GC content of 56.5% and 647 predicted open reading frames (Figure 1). Interestingly, plasmid typing revealed that this plasmid does not belong to any known replicon type. The plasmid harbors a wide array of resistance genes, including β-lactam resistance genes (*bla*_AFM-1_, *bla*_OXA-246_, and *bla*_CARB-4_), sulfonamide resistance gene (*sul1*), quinolone resistance gene (*qnrVC1*), aminoglycoside resistance genes (*aac(6′)-II* and *ant(2″)*), trimethoprim resistance gene (*dfrA27*), chloramphenicol resistance genes (*catB11* and *cmlA8*), rifampin resistance gene (*arr-3*), macrolide resistance genes (*msr*(E), *mph*(E), and *mph*(F)), and bleomycin resistance gene (*ble*_MBL_).

BLAST analysis revealed that no plasmids identical to the *bla*_AFM-1_-positive plasmid (pAFM-PA19-3158) were found in the NCBI database. However, the plasmids pOZ176 (74% coverage, 99.95% identity) and pWTJH17 (79% coverage, 99.99% identity) exhibited the highest similarity to pAFM-PA19-3158. Both of these plasmids were derived from human-associated *P. aeruginosa*. Compared with the other two plasmids, plasmid pAFM-PA19-3158 carries additional resistance genes (*ant(2″)-Ia* and *mph*(F)), suggesting its capacity to continuously acquire resistance determinants and evolve. This poses a further challenge to clinical treatment options by limiting the effectiveness of available antibiotics.

### Phylogenetic evolution of *bla*_AFM_-positive genomics in the NCBI database from China

To further investigate the genomic epidemiological distribution of *bla*_AFM_-positive *P. aeruginosa* isolates in China, we retrieved all *P. aeruginosa* genome data from the NCBI database and selected *bla*_AFM_-positive *P. aeruginosa* isolates of Chinese origin for further analysis in combination with data from this study (Supplementary Table S2). A total of 33 eligible isolates were identified from the database. Except for two isolates with unknown sources, the remaining isolates were all derived from human samples. These isolates were distributed across five different regions of China, with the earliest detected in 2017. The *bla*_AFM_-positive *P. aeruginosa* isolates have been consistently reported between 2017 and 2023, indicating their persistent presence in clinical settings over time.

**Figure 2:**
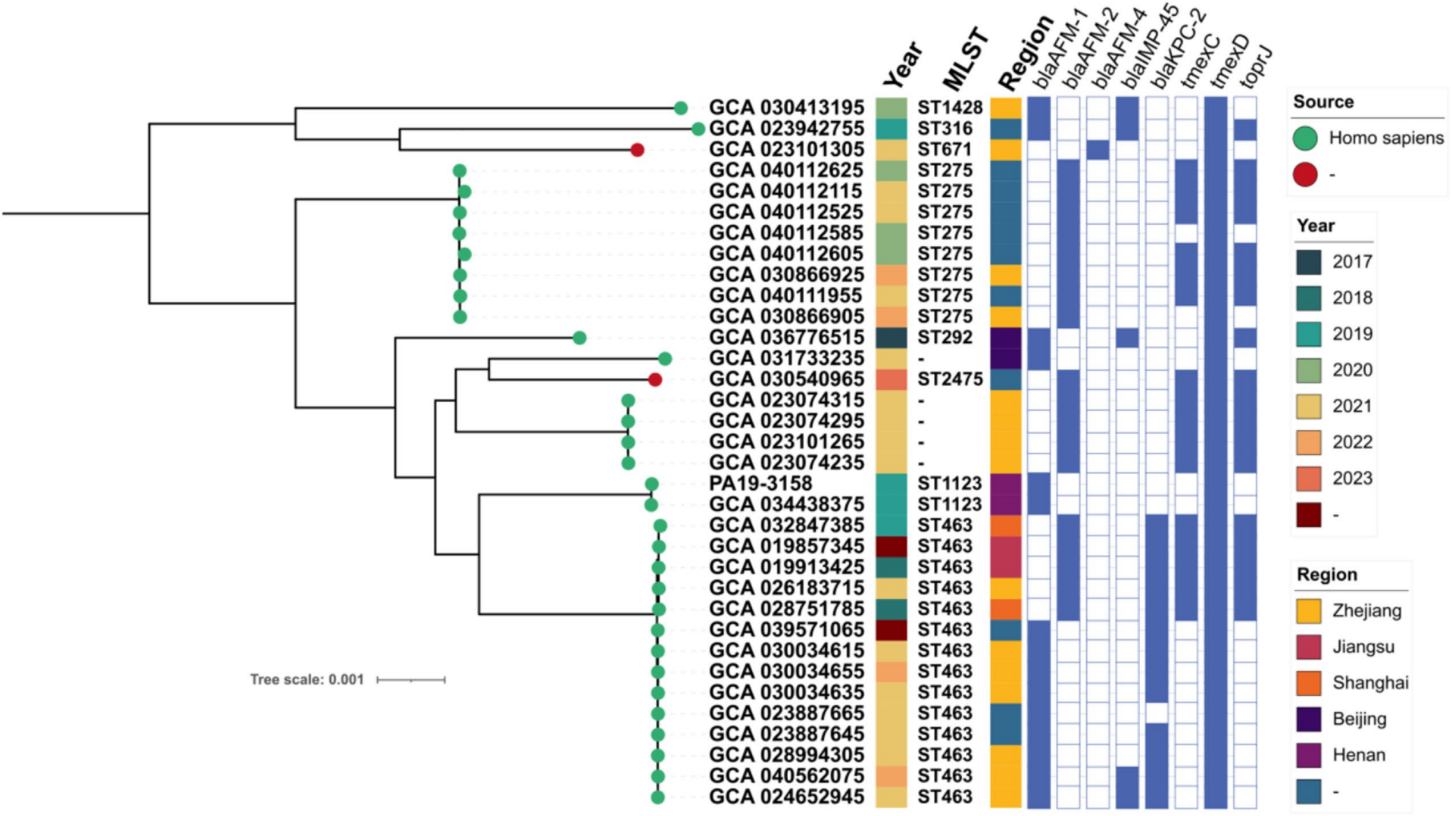
Phylogenetic tree of 34 *bla*_AFM_-positive CRPA strains from this study and the NCBI database. Resistance genes are indicated by squares: solid square indicates positive; hollow square indicates negative.

To investigate the evolutionary relationships of *bla*_AFM_-positive CRPA in China, we constructed a phylogenetic tree combining *bla*_AFM_-positive CRPA isolates from this study with those retrieved from the NCBI database (Figure 2). We observed a high genetic similarity (SNP number < 100) among *bla*_AFM-1_-positive strains from different regions, indicating a potential risk of clonal transmission (Supplementary Table S3). Additionally, the *bla*_AFM_ genes in these isolates were predominantly *bla*_AFM-1_ (*n* = 15) and *bla*_AFM-2_ (*n* = 18), with only one strain carrying *bla*_AFM-4_. MLST analysis classified these isolates into eight known STs, with ST463 being the most prevalent (*n* = 14), followed by ST275 (*n* = 8). Notably, *bla*_AFM_-positive ST463 strains were detected in all years except 2017 and 2023, suggesting its sustained presence in clinical settings over time.

### Distribution of ARGs in *bla*_AFM_-positive strains

Resistance gene analysis revealed that *bla*_AFM_-positive *P. aeruginosa* isolates harbored a wide range of ARGs. Across different years, 10 shared ARGs were identified among the isolates, with those from 2021 carrying the highest number of unique ARGs (*n* = 9), followed by 2019 (*n* = 5). In addition to the *bla*_AFM_ gene, other carbapenem resistance genes such as *bla*_IMP-45_ (*n* = 5) and *bla*_KPC-2_ (*n* = 13) were also detected in several isolates. Notably, ARGs including *aph(3′)-IIb* (34/34), *fosA* (34/34), *sul1* (34/34), *tmexD* (34/34), *ble*_MBL_ (34/34), *catB7* (34/34), and *bla*_PDC-374_ (32/34) were identified in the majority of isolates. In contrast, genes such as *bla*_TEM-1_, *bla*_OXA-494_, and *bla*_PDC-172_ were detected in only a single isolate. This highlights both the conserved and variable resistance profiles of *bla*_AFM_-positive *P. aeruginosa* across different years.

### Correlation analysis of ARGs, virulence factors, insertion sequences and plasmid replicons

**Figure 3:**
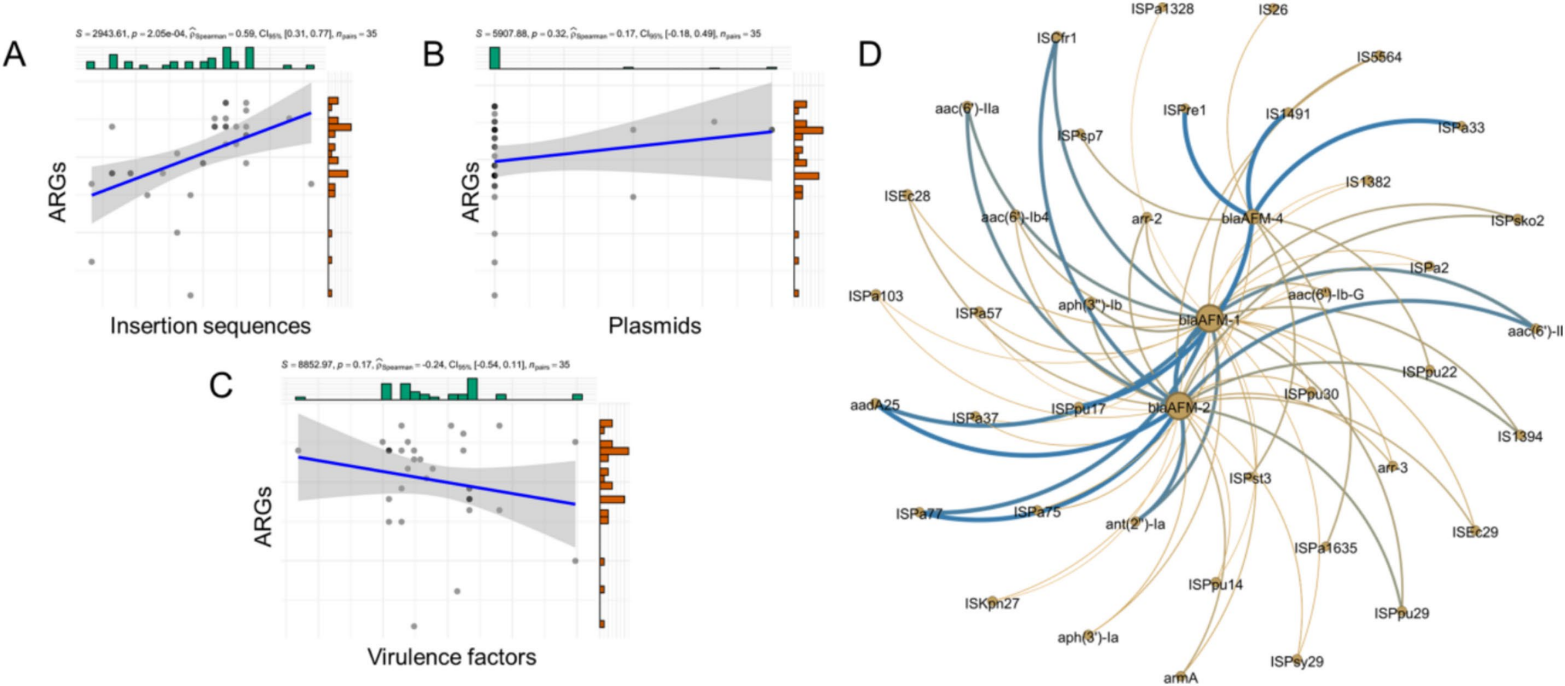
Correlation between ARGs, plasmid replicons, insertion sequences and Virulence genes. **(A–C)** The correlation of counts of ARGs, plasmid replicons, insertion sequences and VFs detected from genomes of CRPA. **(D)** The correlation of *bla*_AFM_ with other ARGs and insertion sequences. The nodes represent ARGs or insertion sequences. Connections between nodes indicate that they are related. Bluer colors and thicker lines represent stronger positive correlations. The yellower the color and the thicker the lines represent the stronger the negative correlation. All associated genes in the figure had *p* values less than 0.05.

In total, 68 ARGs, 3 plasmid replicons, 67 insertion sequences, and 333 virulence factors were identified in the *bla*_AFM_-positive *P. aeruginosa* isolates. To investigate the relationship between ARGs, insertion sequences, plasmid replicons, and virulence factors in these isolates, a correlation analysis was performed (Figures 3A–C). The linear association analysis indicated that the number of ARGs in *bla*_AFM_-positive isolates showed a positive correlation with insertion sequences (rSpearman = 0.31, *p* < 0.001). However, no significant correlations were observed with plasmid replicons or virulence factors (|rSpearman| < 0.3).

To further examine the relationship between ARGs and insertion sequences, a network graph was constructed (Figure 3D). The results revealed that *bla*_AFM-2_ exhibited strong positive correlations with IS*Cfr1*, *ant(2″)-Ia*, and *aac(6′)-IIa* (*R* > 0.8, *p* < 0.05). Similarly, *bla*_AFM-1_ was positively correlated with *aph(3″)-Ib*, IS*5564*, and *aac(6′)-Ib-G* (*R* > 0.5, *p* < 0.05). In contrast, no ARGs showed significant correlations with *bla*_AFM-4_, although it displayed positive correlations with insertion sequences IS*Pa1635*, IS*Ppu22*, and IS*Psp7* (*R* > 0.5, *p* < 0.05).

## Discussion

In 2021, Lin et al. identified AFM-1 (*Alcaligenes faecalis* metallo-β-lactamase-1), a novel metallo-β-lactamase (MBL), as a new variant of NDM-1 through molecular and functional characterization (Zhang et al., 2021). Phylogenetic analysis demonstrated that AFM-1 shares 86% amino acid sequence homology with NDM-1. However, due to the low detection rate of the *bla*_AFM_ gene in *P. aeruginosa*, systematic studies on *bla*_AFM_-positive strains remain scarce (Chen et al., 2022; Zhang et al., 2023). To address this knowledge gap, this study conducted long-term surveillance of *bla*_AFM_-positive strains in hospitals in Henan Province, China. Using genomic approaches, we characterized the features of a *bla*_AFM_-positive strain isolated from Henan. Besides, we further analyzed the genomic characteristics of all *bla*_AFM_-positive CRPA isolates reported in China.

Previous studies have indicated that *bla*_KPC_-producing CRPA accounts for approximately 40% of clinical CRPA infections in certain regions, with ST463 identified as the predominant *bla*_KPC_-positive clone (Hu et al., 2021). Importantly, ST463 has emerged as a major CRPA lineage responsible for bloodstream infections and is associated with significantly higher mortality rates compared to non-ST463 CRPA infections (Zhang et al., 2023). Our findings revealed that ST463 is the dominant clone among *bla*_AFM_-positive CRPA in China, with approximately 38% of these isolates co-harboring the *bla*_KPC-2_ gene. The coexistence of both enzymes may confer resistance to nearly all β-lactam antibiotics severely limiting treatment options, increasing healthcare costs, and elevating patient mortality rates. The potential spread of these multidrug-resistant and highly pathogenic strains poses a significant risk of clinical treatment failure, underscoring the urgent need for continuous surveillance of ST463 *bla*_AFM_-positive CRPA strains.

The *bla*_AFM_ gene has been detected in various bacterial genera, but it is relatively rare in *P. aeruginosa* (Li et al., 2022; Fang et al., 2023). However, due to its ability to spread via plasmids and other mobile genetic elements, it warrants close attention (Chen et al., 2022; Zhou et al., 2023). This study identified a novel, large plasmid carrying the *bla*_AFM_ gene, which harbors a greater number of resistance genes compared to other similar plasmids. This suggests that plasmids carrying the *bla*_AFM_ gene may continuously acquire additional resistance genes from external sources, driving their evolution. However, the plasmid is relatively large and lacks an oriT site, which may explain its inability to transfer. Moreover, we observed that these *bla*_AFM_-positive CRPA strains not only harbor the *bla*_AFM_ gene but also carry multiple important resistance genes. Notably, over time, the variety of resistance genes carried by *bla*_AFM_-positive CRPA strains has steadily increased.

In studies of human and animal microbiomes, a considerable proportion of plasmids have been found to be untypeable using existing replicon-based classification systems. For instance, a study involving donors from China and the United States identified 5,372 plasmid-like clusters (PLCs), of which only 18.9% (155 clusters) could be assigned to one of the 37 known replicon types; the remaining 81.1% were untypeable (Yang et al., 2023). These untypeable plasmids may possess novel replication mechanisms or exhibit highly divergent replication origin sequences. Importantly, they often carry multiple antimicrobial resistance genes and evade detection by current replicon-based surveillance frameworks (Lassalle et al., 2023). As a result, conventional plasmid epidemiology may fail to capture their distribution and contribution to resistance dissemination. The prevalence of untypeable plasmids highlights the remarkable genomic plasticity of bacteria and underscores their relevance in tracking resistance spread, improving monitoring strategies, and designing novel genetic vectors.

Despite the geographic diversity of the *bla*_AFM_-positive CRPA isolates, evolutionary analysis revealed only minimal SNP diffe rences between isolates from different regions, indicating a potential risk for the transmission of resistance genes due to population movement. Notably, we observed that the *bla*_AFM_ gene often coexists with the *bla*_KPC_ and *tmexCD-toprJ* genes. Previous studies have shown that the *tmexCD-toprJ* efflux pump system can mediate resistance to tigecycline. As tigecycline is considered a last-resort antibiotic for treating infections caused by carbapenem-resistant bacteria, the emergence of *tmexCD-toprJ*-positive carbapenem-resistant strains compromises the efficacy of tigecycline, severely limiting therapeutic options and increasing the risk of clinical treatment failure (Zhang et al., 2022; He et al., 2019).

Insertion sequences and plasmids are crucial in mediating the spread of resistance genes (Li et al., 2024). To further investigate their roles in *bla*_AFM_-positive CRPA isolates, we conducted a correlation analysis. We found that, unlike other carbapenem-resistant bacteria, plasmids play a limited role in the acquisition of foreign resistance genes in *bla*_AFM_-positive CRPA (Li et al., 2024). The acquisition and spread of resistance genes appear to be primarily associated with insertion sequences, such as IS*Cfr1*, IS*Pre1*, and IS*1491* (Li et al., 2025). Additionally, we observed significant positive correlations between the *bla*_AFM_ gene and multiple other resistance genes, which may result in a multidrug-resistant phenotype. This highlights the potential risk and warrants increased vigilance in managing these strains.

We acknowledge several limitations in the current study. First, only a single *bla*_AFM_-positive CRPA isolate was recovered, limiting the sample size and broader representativeness. Second, our analysis focused solely on *bla*_AFM_-positive CRPA strains from China, without including genomic comparisons with globally reported *bla*_AFM_-positive CRPA isolates.

## Conclusion

This study systematically analyzed the genomic epidemiological characteristics of *bla*_AFM_-positive CRPA in China, revealing the resistance gene profiles and evolutionary traits associated with these isolates. *bla*_AFM-1_ and *bla*_AFM-2_ were identified as the predominant *bla*_AFM_ variants, and *bla*_AFM_-positive CRPA strains from China were frequently found to harbor multiple critical resistance genes and exhibit diverse ST types. The resistance genes in *bla*_AFM_-positive CRPA strains from different regions demonstrated potential for interregional dissemination. The emergence of *bla*_AFM_-positive CRPA across multiple provinces in China highlights the urgent need for stringent monitoring and the implementation of appropriate measures to mitigate the future threats posed by these strains.

## Data Availability

The datasets presented in this study can be found in online repositories. The names of the repository/repositories and accession number(s) can be found in the article/Supplementary material.

## Footnotes

^1^ https://github.com/tseemann/snp-dists
